# Do Camels (*Camelus dromedarius*) Need Shaded Areas? A Case Study of the Camel Market in Doha

**DOI:** 10.3390/ani11020480

**Published:** 2021-02-11

**Authors:** Martina Zappaterra, Laura Menchetti, Leonardo Nanni Costa, Barbara Padalino

**Affiliations:** Department of Agricultural and Food Sciences, University of Bologna, Viale Fanin 46, I-40127 Bologna, Italy; martina.zappaterra2@unibo.it (M.Z.); laura.menchetti@unibo.it (L.M.); leonardo.nannicosta@unibo.it (L.N.C.)

**Keywords:** one-humped camel, behavior, shade, space allowance, ethogram, rumination, sternal recumbency, stereotypic behavior, welfare

## Abstract

**Simple Summary:**

Scientific knowledge concerning dromedary camel behavior and welfare is still limited. To date, providing pens with adequate shaded areas is not regulated in camel husbandry. The objectives of this study were to document whether dromedary camels have a preference for shade and describe how their behavior would change depending on the presence of shade in pens with different animal densities. Analyzing the behavior of camels kept at a permanent market in Doha, we found they had a preference for shade, and adequate shaded areas seemed to exert a positive effect on their behavioral repertoire. Camels in shade expressed more natural behaviors such as lying in sternal recumbency and ruminating, while those in the sun showed more walking and standing. Limited space allowance, instead, seemed to affect camel welfare, increasing the expression of stereotypic behavior (i.e., pacing). Overall, the results of this pilot study suggest that provision of adequate shaded areas could safeguard camel wellbeing under extremely hot conditions.

**Abstract:**

This study aimed at documenting whether dromedary camels have a preference for shade and how their behavior would change depending on the presence of shade and variable space allowance. A total of 421 animals kept in 76 pens (66 with shelter (Group 1), and 10 without shelter (Group 2)) at the camel market in Doha (Qatar) were recorded for 1 min around 11:00 a.m. when the temperature was above 40 °C. The number of animals in the sun and shade and their behaviors were analyzed using an ad libitum sampling method and an ad hoc ethogram. The results of a chi-square test indicated that camels in Group 1 had a clear preference for shade (*p* < 0.001). The majority of Group 1 camels were indeed observed in the shade (312/421; 74.11%). These camels spent more time in recumbency and ruminating, while standing, walking, and self-grooming were more commonly expressed by the camels in the sun (*p* < 0.001). Moreover, locomotory stereotypic behaviors (i.e., pacing) increased as space allowance decreased (*p* = 0.002). Based on the findings of this pilot study, camels demonstrated a preference for shade; shade seemed to promote positive welfare, while overcrowding seemed to trigger stereotypy and poor welfare. Overall, our preliminary results are novel and provide evidence that shaded areas are of paramount importance for camel welfare. Further research, involving designed studies at multiple locations is needed to confirm these results.

## 1. Introduction

The dromedary camel (*Camelus dromedarius*, one-humped camel or Arabian camel) is one of the most important natural resources in Africa and the arid lands of the Middle East and Western Asia [1,2]. In 2018, Africa held more than 86% of the world’s stocks of camels [1]. In these countries, the dromedary camel plays a role of great economic and social importance, as it is used for transport, racing, tourism, and first of all, provides food of high nutritional value (meat and dairy products), wool and leather, where the common ruminant livestock species (cattle, sheep and goat) cannot be efficiently reared for the same purposes [2]. Although heat stress and drought strongly reduce livestock productivity, health, and fertility [3], camels have developed a unique physiological adaptation to heat stress [4] and efficient milk production, even after one week of water deprivation [5]. Camels’ ability to cope with extreme heat stress and drought is seen as an opportunity in adapting to climate change [6]. Consequently, camel rearing is growing worldwide and camel husbandry, physiology, and genetics are receiving increasing interest from the research community [4,7,8,9]. Despite this growing interest, scientific knowledge regarding camel behavior and welfare is still scarce. Few studies have been carried out to assess the effects of housing systems in camels [9,10,11]. In particular, confinement and lack of social interaction were found to affect the well-being of these animals [9,10]. Male dromedary camels housed in single stalls had higher blood cortisol levels and showed more stereotypic behavior than the subjects who were housed in open paddocks and interacted with females [9,10]. The freedom of movement and the olfactory contact with females permitted the expression of sexual behaviors and increased testosterone levels in male camels [11]. The literature therefore suggests that management choices, including space allowance, are critical for camel welfare [12].

Several studies exist on the beneficial effects exerted by shaded areas on the welfare and production efficiency of other livestock species [13,14,15,16]. Horses were proven to prefer shade in hot and sunny environments, and perform more walking and foraging behavior in shaded areas [14,16]. It was also found that horses kept in the sun showed reduced feed intake and movement, while increased amounts of water drunk and time spent close to a drinking point [17]. Thus, shade permitted greater expression of natural behaviors in horses. Similarly, in dry conditions, shade was found to have a positive effect on the behavior of water buffaloes, which spent more time grazing and ruminating when allotted in shaded paddocks than in unshaded ones [18]. The presence and amount of shade were proven to affect cow behavior too [13,15]. The latter studies indicated that cows managed on pastures prefer shaded areas, spending more time in the shade as environmental heat load increased, highlighting the importance of providing an effective shaded area to cows reared in extensive systems [13,15]. Furthermore, when a higher amount of shade per cow was provided, the time cows spent more time lying down [15]. Smaller sized shaded areas and increased heat load were also associated with increased probabilities of observing aggressive interactions among cattle [15]. When more space was given, cattle were, instead, less likely to display aggressiveness, sharing the resource, rather than competing for it [15]. In agreement with these observations, in hot and dry conditions, pigs were found to prefer shade, where they spent about 80% of their time between 7:00 a.m. and 3:00 p.m. [19].

There is considerable evidence that shade prevents the deleterious effects of heat stress on health and production in domestic livestock [20,21], and that cattle, horses, buffaloes, and pigs prefer shaded areas [13,14,15,16,18,19,21]. This evidence has been useful for implementing a number of Code of Practices for farm animal welfare [22,23,24] and shade is currently recommended by the World Organization for Animal Health (OIE) Terrestrial Animal Health Code [25]. Despite camel-related research noticeably increasing, the parallel evolution of specific welfare laws has been limited [26], and research outputs may encourage lawmakers to develop official regulations. The Australian Code of Practice for camels reports that dromedary camels store their latent heat during the day and shed it at night, but once the body temperature exceeds 40 °C they will commence sweating, thus, efficient ventilation and protection from sunlight are essential [22]. Since it has been proven that higher temperatures and levels of humidity are risk factors for heat-stroke in camelids [27], studies investigating factors mitigating welfare hazards in dromedary camels, such as shaded areas, are crucial. However, there are no scientific studies regarding the effect of shaded areas on camel behavior and welfare.

The main hypothesis of this study was that dromedary camels would show a preference for shade in a hot and sunny environment and the second hypothesis was that shaded areas would have a positive effect on camel behavior. Consequently, this study aimed to document whether dromedary camels have a preference for shade and describe how their behavior changes depending on the presence of shade in pens with different animal densities.

## 2. Materials and Methods

### 2.1. Study Site and Animals

This study was conducted at the dromedary camel Market in Doha (Qatar), from the 10 to the 17 of September 2019. A total of 76 pens with 421 animals were considered for the present study. In each pen, there was a different number of animals (from 1 to 22; median = 4) of both sexes and different ages. The animals came from farms located in Qatar and in other countries (i.e., Sudan, Oman) and were kept at the market for different purposes (i.e., meat, milk, breeding or race). The animals were group-housed mainly based on their age and purpose. The animals for meat and racing were mainly juveniles who were kept in large groups (*n* > 15). Animals for milk production were adult females with calves, usually kept in small groups (*n* < 5). Animals for breeding were mainly adult females, kept in medium-sized groups (*n* < 15). Only in one paddock, a bull was kept with the breeding females. The other bulls (*n* = 3) were kept individually and brought to the females only for mating. Since they were tethered, their pens were not included in the study.

Based on the information reported by the caretakers [28], the animals were familiar with each other. The time they were kept at the market varied based on the purpose of the animals, with breeding and milking females being kept for longer periods of time than meat and racing animals. On the whole, the majority of the camels were in good health status but only 23% had an optimal Body Condition Score (BCS, i.e., 3 on a scoring scale from 1 to 5 [29]).

Among the 76 pens, 66 had a shelter creating a shaded area in the pen (Group 1), while 10 had no shaded area at all (Group 2). The measures of the pens and shelters were taken with a measuring tape. In order to not affect the behavior of the penned camels, the measurements of the facilities were taken on a different day to when behavior was recorded. Pens were rectangular or square-shaped; in some cases, various materials such as broken furniture, were stored in the pens, thus only the area available to the camels was measured. In each pen, the numbers and shapes of feeding and drinking points varied. Each pen had a number of feeding points ranging from 1 or 2 (48 pens) to more than 2 (27 pens). One pen was not provided with feeding points and the animals were ground-fed. Feeding points were mainly located in the sun (63 pens). The number of drinking points ranged from 0 (7 pens) to 2 or more (5 pens), and most of them were located in the sun (73 pens). The animals kept in the pens with no drinking points were watered using buckets either daily or every two days [28].

### 2.2. Animal Behavior and Environmental Parameters

As pens were surrounded by dirt roads and the camels were used to cars, the operator recorded the videos from inside a car parked in front of the pen. Camels in each pen were recorded for 60 s by the same operator (BP). Recordings were made throughout the eight days of sampling, about 9–10 pens per day, starting from the same time of the day (11:00 a.m.) and finishing around 11:30 a.m. Videos were taken using a Legria HF M46 camera (Canon Inc, Ōta, Tokyo, Japan) and durations were tracked with a stopwatch (Swatch, Swiss, Europe). Videos were recorded with a simultaneous commentary on the recording date and time, the number of camels in the pen, and pen conditions. After recording the behavior, the water temperature was taken with a digital thermometer (Mabis, Briggs Health Care, West Des Moines, IA, USA) and environmental parameters (i.e., temperature, humidity, and wind speed) were measured at the level of the camels’ nose, using a weather station (Testo 410-2, Testo Spa, Milan, Italy). The water quality was also assessed for each drinking point and half of the pens had dirty (25 pens) or partially dirty (17 pens) water.

The videos were analyzed by two observers that were previously trained in analyzing the behavior of dromedary camels in videos recorded for other studies. After training, 20 videos out of the 76 were analyzed by both observers to test interobserver reliability, expressed as percentage agreement. Interobserver reliability ranged between 95 and 99% agreement for the behaviors measured, such as duration and frequency, respectively. The remaining 56 videos were then equally divided between the two observers and analyzed. The first step of the behavioral analysis was to count the number of animals in the shade and sun in each pen. An animal was considered to be in the shade when it had at least two legs and more than half of the body in the shaded area. For Group 1, the observers also annotated if there were camels in the sun due to there being no shade available (i.e., the whole shaded area was occupied by other animals). The behavior was then analyzed by an ad libitum sampling method [30] using the ethogram adapted from Padalino et al. [9] reported in Table 1. The duration of the behavioral states (i.e., feeding, rumination, drinking, walking, standing, recumbency, self-grooming, pacing in circles/on a line, self-biting) and the frequency of the behavioral events (i.e., positive interaction, aggressive interaction, vocalization, bar-mouthing, head-shaking) were noted. Only the locomotory states (i.e., walking, standing, recumbency) were considered mutually exclusive. Pacing was distinguishable from walking as camels showing this stereotypy had faster and recursive moves, without a clear motivation. For each behavior, it was annotated whether the camel was showing it while in the shade or in the sun.

### 2.3. Data Handling

The pen measurements and the behavioral data were reported in an Excel file. For each pen, the space available for the animals was calculated by subtracting the space unavailable for the animals from the whole area of the pen. Thus, the camel space allowance per pen (m^2^/camel) was calculated by dividing the space available by the number of camels.

Behavior durations and frequencies were normalized by multiplying them by the number of animals showing that particular behavior and then dividing it by the number of animals in the sun or in the shade (i.e., the duration/frequency of the behaviors expressed by camels in the sun was normalized considering the number of animals in the sun and the duration/frequency of the behaviors noted in camels in the shade was normalized considering the number of animals in the shade).

### 2.4. Statistical Analyses

The descriptive statistics were initially performed on all behavioral data, and then stratified by group and location of the camels (i.e., Group 1 sun, Group 1 shade, and Group 2 sun). The relative proportion (%) of the mutually exclusive locomotory behaviors was calculated, stratifying the camels by group and location, and the proportions are graphically displayed using pie charts. Descriptive statistics, in addition to diagnostic graphs, were also used to verify data distribution. Data were not normally distributed.

Inferential statistics were carried out on the behavioral data of camels kept in pens in Group 1 (*n* = 66) and included both univariate and multivariate approaches. Camel preference for shade was evaluated with a chi-square test comparing the observed and expected numbers of camels in shade. As the size and space allowance per camel varied among the pens, the expected number of camels in shade (size of the shaded area (m^2^)/space allowance per animal in the pen (m^2^/camel)) was calculated for each pen. The expected number of camels in shade was therefore estimated assuming that there was no preference (i.e., the number in shade is equal to the proportion of shade available).

In order to take into account the possible effect of different space allowances on camels’ behaviors, univariate Generalized Linear Models (GLMs) using the Poisson distribution [31] were used to test whether location (i.e., sun and shade) and decreasing space allowance had an effect on the behaviors of Group 1 camels. The GLMs were performed with the behaviors as dependent variables, the location as a fixed effect, and the space allowance as the covariate. The results of the associations between location and behaviors were reported as means ± standard errors (SE), while the covariate effect of space allowance was reported as the parameter estimate (b).

Finally, for Group 1, a discriminant analysis (DA) was performed to identify the linear combinations of behaviors (discriminant function (Df)) that best discriminate the location of the camels (shade vs. sun). The DA classified the behaviors according to their importance in this discrimination. The behaviors were included in the DA as independent variables, while location (sun and shade) was included as a group variable. Mahalanobis distance was used to verify multivariate normality and identify the presence of multivariate outliers. The relative importance of each behavior in classifying the camels’ location was evaluated by using Wilks’ lambda (the smaller the Wilks’ lambda score, the more important the variable to the Df) and by the discriminant loadings (correlations between each independent variable and the discriminant scores associated with the Df) [32,33]. Moreover, the discriminant scores were calculated as a weighted linear combination of the behaviors. The centroids indicated the mean discriminant scores of Df for camels in shade and the sun. They were used to establish the cutting point for classifying samples during the cross-validation. The leave-one-out cross-validation was performed as an index of Df performances, calculating the probability for each item to be accurately classified in the correct location [32,33].

Statistical analysis was performed using SPSS 25.0 (SPSS, an IBM Company, Chicago, IL, USA), and R environment [34]. *p* < 0.05 were considered statistically significant.

## 3. Results

The temperature ranged from 40.40 °C to 47.20 °C, with an average temperature of 43.46 ± 1.91 °C. Relative humidity ranged from 16.50 to 40.40%, with a mean value of 27.03 ± 8.09%. The average wind speed was 3.18 ± 2.92 km/h, with the observed minimum value of 0 km/h and a maximum of 9.90 km/h. The water temperature ranged from less than 32 °C to more than 43 °C, with an average temperature of 36.75 ± 2.86 °C.

The pen size ranged from 26.41 to 254.80 m^2^ (on average 138.74 ± 52.92 m^2^). The average of unavailable space per pen due to various materials was 7.13 ± 13.10 m^2^, ranging from 0 to 74.46 m^2^. The actual space allowance varied from 4.5 m^2^ per animal to 100 m^2^ per animal. Pens with shelters (Group 1) had shaded areas with a size ranging from 5.0 to 71.1 m^2^ (on average 24.97 ± 17.31 m^2^). The shaded areas covered between 4.40 to 50.81% of the total space of the pen available for the animals, with an average size of the shade covering 24.33 ± 11.33% of the pen.

### 3.1. Descriptive Statistics of the Behaviors Observed in Group 1 and 2

Recumbency, standing, rumination, and feeding were the behaviors most commonly observed and with longer durations. For the whole camel sample, recumbency was the behavior observed for the longest period, with an average duration of 38.9 s. Sternal recumbency was often observed in the shade, and in pens without shelter (Group 2 sun), where camels often rested near the fences (Figure S1). Lateral recumbency was only noticed in the shade (Figure S2) in one camel which was kept in an individual pen of 90 m^2^ with a shaded area of 15 m^2^. In general, the average duration spent standing was 16.2 s and the average duration spent feeding was 3.6 s. Although the feeding points were partly located in the sun, most camels tended to eat in the shaded area (Figure S3a,b). The average rumination time was 6.9 s. Among the stereotypic behaviors, pacing in circles/on a line was the only behavior noticed during the video analysis, with three camels showing this stereotypy in Group 1 sun and two camels in Group 1 shade. Drinking was never observed. Descriptive statistics of the observed behavioral data stratified by Group and location (i.e., Group 1 sun, Group 1 shade, and Group 2 sun) are reported in Table S1.

**Figure 1:**
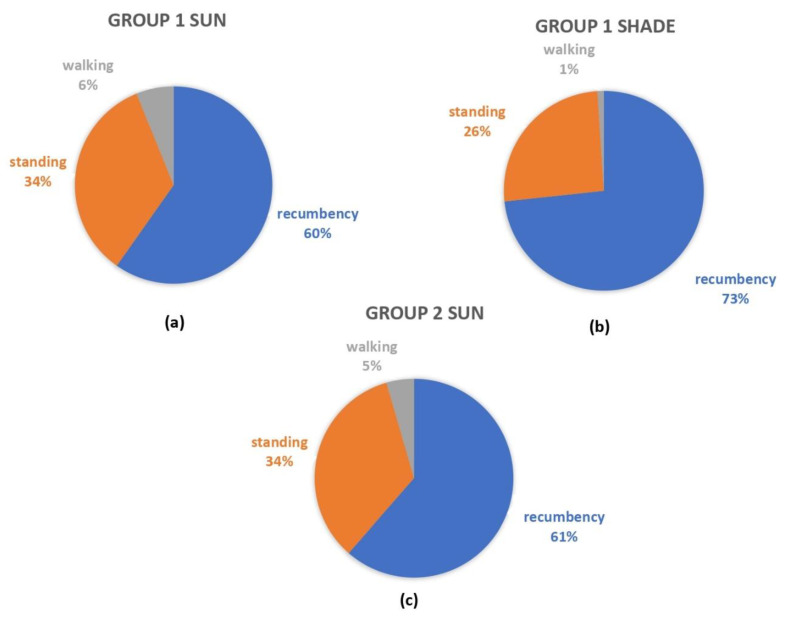
Relative proportions of the mutually exclusive locomotory behaviors shown by dromedary camels kept in different type and locations of pens at the camel market in Doha: (**a**) in the sun of the pens with shelter (Group 1 sun); (**b**) in shaded areas of the pens with shelter (Group 1 shade); (**c**) in the sun of pens without shelter (Group 2 sun).

Figure 1, showing the proportions of the mutually exclusive locomotory behaviors stratified by group, indicated that camels in the shade spent triple the amount of time in recumbency in comparison to standing, while those in the sun spent twice the time in recumbency in comparison with standing. Camels in the sun walked more than those in the shade.

### 3.2. Statistical Analysis of the Behavioral Data of Group 1

Among the 66 pens of Group 1, 25 had animals in the sun as the shade was completely occupied by other camels (25/76; 37.88%). The median number of animals in the sun due to there being no other shade available ranged from one to eight, with a median number of three. Among the 421 camels, 312 animals were observed in the shade (312/421; 74.11%) and only 109 in the sun (109/412; 25.89%).

#### 3.2.1. Chi-Square Test

**Figure 2:**
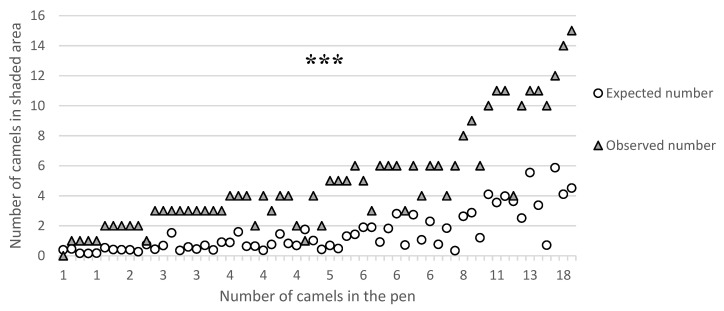
Comparison of the expected and observed number of dromedary camels in the shade in Group 1 pens (with different sizes and number of animals housed) at the camel market in Doha: comparison of the expected (white circles) and observed (grey triangles) numbers of dromedary camels located in shaded areas. *** means chi-square *p* < 0.001.

**Figure 3:**
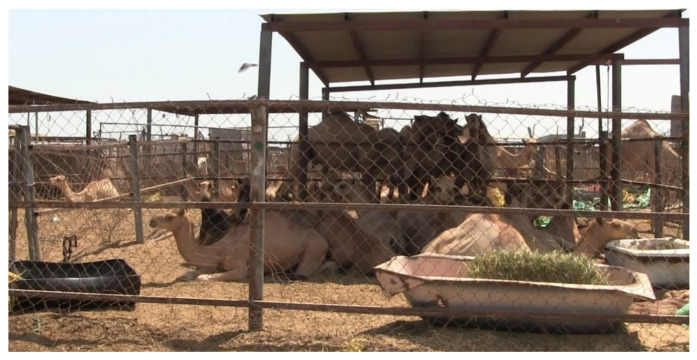
Frames of videos recorded at Doha market with dromedary camels crowded under the shelter, only one animal is resting with almost all its body in the sun because the shade is not available.

Figure 2 reports the comparison between the number of dromedary camels observed and expected in the shaded areas. The chi-square test indicated that the number of camels observed in the shaded areas was higher than the expected number (*p* < 0.001), and as the total number of dromedary camels kept in the same pen increased, the preference for shade became more evident (Figure 3).

#### 3.2.2. Univariate Generalized Linear Models (GLMs)

**Figure 4:**
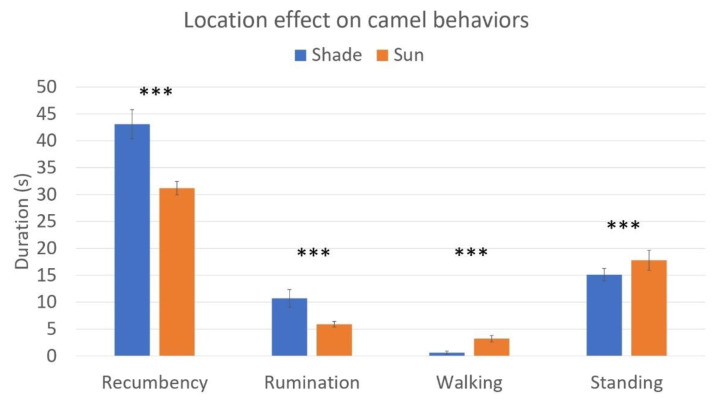
The effect of location (Group 1 shade vs. Group 1 sun) on the duration of recumbency, rumination, walking and standing in camels kept in pens with a shelter (Group 1 shade and Group 1 sun). The means and standard errors are graphically presented. *** means *p* < 0.001.

Figure 4 shows the effect of camels’ location on the duration that each observed animal spent in recumbency, ruminating, standing, and walking in the 66 pens of Group 1. Camels in the shade showed more recumbency (*p* < 0.001) and rumination (*p* < 0.001), while camels in the sun showed more walking and standing (*p* < 0.001). Besides the behaviors reported in Figure 4, camels’ location was also significant for self-grooming (*p* < 0.001), which was more expressed in the sun. Standing was associated with both locations (*p* < 0.001) and space allowance, with camels spending more time standing in the sun as space allowance decreased (b = −0.003; *p* = 0.005). Feeding was also negatively associated with space allowance, with camels spending longer periods of time feeding as space allowance decreased (b = −0.017, *p* = 0.001). Finally, space allowance was negatively associated with stereotypic behavior, namely pacing in circles/on a line; this stereotypic behavior increased as space allowance decreased (b = −0.029, *p* = 0.002).

#### 3.2.3. Discriminant Analysis (DA)

**Figure 5:**
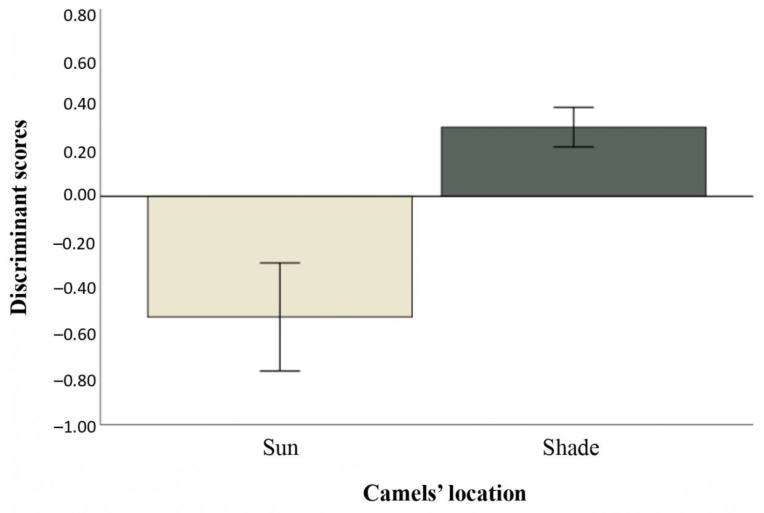
Means and standard errors of the discriminant scores identified for the behaviors of dromedary camels in the sun and shaded areas of pens in the Doha market. Walking, recumbency, and vocalization were the behaviors that significantly contributed to the discriminant function.

Table 2 reports the discriminant loadings, Wilks’ Lambda, and the significance of the F test for each variable included in the DA. Feeding, aggressive interaction, and pacing in circles/on a line were not included as they had low loading values (<|0.050|). Moreover, seven observations were discarded as they were multivariate outliers according to the Mahalanobis distance. Walking, recumbency, and vocalization (*p* < 0.050) were the behaviors that mostly differed according to the location (i.e., sun and shade), followed by a tendency for rumination (loadings ≥ |0.400|; Wilks’ Lambda < 0.99; *p* < 0.2). The discriminant scores (Figure 5) and centroids (−0.528 and 0.303 for sun and shade, respectively) showed that walking and vocalization significantly prevailed when the camels were in the sun, while recumbency and rumination prevailed when camels were located in the shaded areas of the pens. The other variables had minor importance in discriminating the location (loadings < |0.400|; Wilks’ Lambda > 0.99; *p* > 0.2). Overall, the DA showed a moderate discriminating ability: the Wilks’ Lambda model was at the verge of significance (*p* = 0.057) and the extracted Df correctly classified 68.9% of the samples.

## 4. Discussion

The present study documented whether dromedary camels have a preference for shade and described how their behavior changed depending on the presence of shade in pens with different animal densities. Our results supported our hypotheses. In this pilot study, the animals chose to be in the shade, occupying all the space available under the shelter instead of being equally distributed in the pen. Our results are novel and seem to suggest that shade has a positive effect on the behavioral repertoire and welfare of camels.

In the present study, dromedary camels showed a clear preference for shade. This was expected based on the literature concerning the preference for shade in other animals. Horses, cattle, water buffaloes, and pigs have a preference for shade [13,14,15,16,18,19,21]. Even though camelids have a higher range for thermoneutrality, they also suffer from heat stress [27]. Dromedary camels use heterothermy to regulate heat gain, and their body temperature fluctuates throughout the day [35]. These fluctuations become greater when associated with dehydration [35], indicating that dehydration challenges the ability of camels to cope efficiently with heat stress. Camels undergoing experimental dehydration and heat stress greatly decreased their food consumption [35], suggesting that these conditions may also hinder the productivity and welfare of these animals. In our study, the temperature of water in the drinking points was often above 32 °C. The presence of such warm water may therefore explain, at least in part, why drinking behavior was never noticed. Providing *ad libitum* fresh water is also of paramount importance in camels, in order to help these animals to cope more efficiently with the heat load. Unlike other livestock species, camels do not dissipate heat through panting [36]; the measurement of which is, therefore, not useful in assessing camel heat stress. Contrariwise, it has been shown that camels dissipate heat mainly through perspiration [35,37]. Their sweating rate greatly increases when the ambient temperature rises above 35 °C [37,38] and relative humidity falls below 20% [37]. Interestingly, skin temperature and sweating rate have been proven to change in camels’ body regions, suggesting that some specific body parts may be potentially more effective in facilitating evaporative heat dissipation [39]. Among them, the hips and hump were recognized as body parts that possibly work as efficient heat-dissipators [39]. In the present study, the temperatures recorded in the sun were above the thermoneutrality zone for camels, suggesting that the animals were forced to thermoregulate in the sun. When they were available, camels, therefore, chose to be under the shelters, avoiding the heat. The motivation for being in the shade was so strong in our camels, that in pens with small-shaded areas, these animals were often stuck under the shelter, in extremely crowded conditions. In the pens where there was not enough shade for all the camels, we often noticed individuals cuddling around the shaded area, in recumbency, with the posterior or the hump in shade and the other body parts in the sun. This observation seems to be consistent with the role suggested for hump and hips in heat dissipation and may indicate that these animals were trying to thermoregulate their body temperature. Based on our findings, a shelter should be provided in each pen where camels are kept over 35 °C. The shaded area projected by the shelter should also be large enough to provide shade for all the camels in the pen and for placing drinking and feeding points. The latter precaution is of paramount importance in places where solar irradiance is particularly high, as it could cause abnormal fermentation in feeding and increase the water temperature.

In our study, the shaded areas seemed to have a positive effect on the behavioral repertoire of the dromedary camels. The animals in shade spent more time resting in recumbency and ruminating than those in the sun. Non-foraging mouth movements in the form of rumination are a key part of the behavioral repertoire of ruminants [40]. The expression of this behavior is coupled with a quiet state of the animal, which is often lying in recumbency. In cattle, rumination occupies 6–8 h per day and can be accompanied by non-REM sleep [40]. In agreement with studies in cattle, the time camels spend ruminating is about 8.3 h per day, peaking in the morning between 9:00 and 11:00 a.m. and in the night between 2:00 and 4:00 a.m. [41]. It seems, therefore, that only camels of Group 1 in the shade could express rumination in line with their natural daily rhythms [11,41]. In domestic ruminants, normal rumination patterns are disrupted in case of distress, diets high in carbohydrates, and stressful events [40]. Similarly, in camels, rumination was found to be less expressed when in distress, such as in animals housed in high-density pens [42]. Despite the limited observation time, the shorter duration of rumination in camels of Group 1 in the sun may be a sign of distress. Concerning locomotory behaviors, camels spent most of their time in recumbency, and, in particular, in sternal recumbency. The relative proportions of recumbency and standing observed in the shade seem to agree with the observations reported in a previous study in camels in Pakistan, where sitting in sternal recumbency was expressed for a period two and a half times longer than that spent standing [43]. Thus, once again, shade seems to promote the expression of natural behaviors. The higher duration of standing expressed in Group 1 camels in the sun may be explained as a way to cope with heat load, as already reported for cows during heat stress [44,45]. This strategy is seen as a cooling response to heat, maximizing the surface area exposed to the environment and increasing the airflow around the body [45,46]. Thus, in absence of shaded areas, standing may be a strategy used by camels to cope with heat stress. Hypothesizing the actual reason why camels in the sun performed more self-grooming may be challenging. This behavior has indeed been used both as a positive and negative indicator of animal welfare in cattle [47], as it can be both a sign of health and vitality or a sign of inadequate farming conditions (dirt and ectoparasite loads). In the present study, the increased self-grooming in camels in the sun may be more likely ascribed to ectoparasites. Some camels had skin disorders such as mange, which is known to become itchier in the sun. Overall, the increased expression of natural behaviors in the shade (such as sternal recumbency and rumination in Group 1 camels in the shade) could be seen as a positive indicator of animal welfare [48]. Shade, therefore, seems to promote a positive welfare state in camels, and adequate shaded areas should be recommended in camel farming.

In our study, the multivariate approach (i.e., discriminant analysis, DA) was used to detect underlying structures in the data set and identify the pattern of behaviors that mostly discriminated the animals in the sun from those in the shade. The results have reinforced the findings of univariate GLMs, confirming the hypothesis that camel behavior changed depending on the presence of the shade. Interestingly, the multivariate analysis suggested that, together with walking, camels in the sun vocalized more. Vocalization in camels comprises a wide set of different sounds, from blubbering vocalizations during rutting behavior [49], to vocalizations as a sign of distress or pain [50]. In dairy cows, increased vocalizations are common during distressing situations [51] and may also be a sign of suffering during heat stress [44]. We were not able to ascertain the cause of the vocalizations in the observed camels, but it is possible to hypothesize that an increased frequency of vocalizations in camels in the sun may be a sign of distress or frustration. This result seems to suggest once again that the lack of a shelter may be a cause of distress in dromedary camels.

Space allowance is a very important parameter in farming. A space allowance of 15 m^2^ is considered adequate for camels [12]. In our observations, the only camel lying in shade was the one resting in a pen provided with a shaded area of 15 m^2^. This result seems to agree with the literature [12], suggesting that this shaded space allowance would permit the animals to lay down and sleep, allowing the camels enough space for stretching during the resting period [52]. In our study, space allowance was negatively associated with feeding, standing, and pacing. Low space allowance was observed in crowded pens, where more animals were observed to eat from the same feeding points. This observation may suggest that feeding points were of insufficient number and/or size in these pens, and camels tended to compete for feeding. As a result, camels lower in the dominance hierarchy may be left in the sun, while the dominant animals were resting in the shaded areas and taking advantage of this situation approaching the feeding point. Animals in crowded pens may, therefore, suffer from higher levels of frustration than those with a higher space allowance, causing an increase in the duration of stereotypic behavior of pacing. Stereotypies indicate that the animals are in a state of distress, suffering from frustration, threat, fear, lack of control over their environment, and lack of stimulation [53,54]. Pacing was first described by Padalino et al. in male dromedary camels kept in individual boxes [9]. Locomotory stereotypies such as pacing have already been described in other animal species in captivity [55,56]. Confinement has been proven to be one of the main stressors increasing the frequency of stereotypic behaviors [57]. Unlike in the study by Padalino et al. [9], camels in the present study did not show other stereotypic behaviors, such as bar-mouthing, self-biting, or head-shaking. This difference may be ascribed to the different types of housing, as in the study by Padalino et al. [9] the camels were single-stabled, while in the present study most of the camels were group-housed, so their behavioral need of expressing social behavior was met [52]. This aspect, together with the fact that the animals in our study were likely more stimulated by the situation outside their pens, may have contributed to the lower expression of other stereotypies. It ispossible, however, that these stereotypic behaviors were not observed in the present study due to the limited duration of the recordings. Stereotypic behaviors have recursive diurnal variations, and in camels, the frequency and duration of stereotypies peaked before and after food distribution [11]. Due to the in-field nature of the present study, we do not know with certainty the time when the different caretakers distributed food. Most of the pens still had forage in the feeding points and some animals were eating at the time of recording. Even though some stereotypies were possibly underestimated, this study reported—for the first time—pacing in camels kept in groups. This evidence seems to suggest that this stereotypic behavior may result from the effect of confinement and overcrowding. These results are consistent with other livestock species; for example, cows showed stereotypic behaviors when in restraint [58], but not in pasture [59]. Our observations seem to indicate that decreasing space allowance is associated with poor welfare conditions in dromedary camels. Consequently, in camel farming, not only the presence of a shelter should be recommended, but also the total and the shaded space allowance should be regulated to safeguard camel welfare in a hot environment. For instance, to mitigate heat stress in cattle, the OIE suggests having an emergency plan including a reduction in stocking density.

The Australian Code of practices in animal welfare for camels recommended the presence of a shelter when temperatures are higher than 40 °C [22], but based on our findings adequate shaded areas should be recommended worldwide. Our findings are useful for all camel industry members who care about the health, productivity, and welfare of these animals. To safeguard camels’ welfare, their behavioral needs have to be met, respecting the paradigms of the Five Freedoms reported in 1992 by the Farm Animal Welfare Council [60]. The five freedoms, indeed, comprises the freedom from discomfort—defined as the animal living in an appropriate environment, including shelter and a comfortable resting area—and the freedom to express normal behaviors; which can be met by providing sufficient space, proper facilities, and company of the animals’ own kind [60]. Our results seem to indicate that shade allows the camels to spend more time resting in recumbency and ruminating, thus permitting the expression of natural behaviors in this animal species. This result suggests that shade promotes positive states in camels, in agreement with the recent extension of the five freedoms model, highlighting the need to also assess the expression of positive behaviors for grading animal welfare [61].

Our results should be interpreted with caution because our field study was limited by several factors. The first limit is that it was impossible to standardize the protocol. The pens at the market were all different sizes, had different space allowances, locations, and numbers of drinking and feeding points. However, we intended to record the normal behavior without altering any factors where the animals were kept, as a change in the environment could have biased the results. The second limit is the short window of observation, which could have caused an underestimation of some behavioral patterns, such as stereotypies. Our choice was due to logistical reasons and that particular time window was chosen to avoid affecting the routine activity of the market, which started early in the morning and stopped around 11:00 a.m. The third limit was that we did not have exact information on the observed camels as regards their age, dominance rank, and temperament, even though these factors could have played a role in the findings. The fourth limit is the lack of recording of important physical (e.g., air temperature in the shade, wind speed in the pen, the surface temperature and reflectivity of the shelter roof, the direct and diffuse solar radiation in the pens) and physiological measurements (e.g., camels’ heart rate, water balance, skin and core temperatures). These measures are of great importance for drawing further evidence and completing our observations. Notwithstanding these limitations, this was the first field behavioral study describing the effects of shaded areas on camel behavior.

## 5. Conclusions

Overall, this pilot study concludes that dromedary camels prefer shaded areas when ambient temperatures are above 40 °C. In the pens with shelters, the camels in the shaded areas demonstrated natural behaviors, such as recumbency and rumination, for longer durations. Based on our preliminary observations, it seems that shade may promote positive welfare, while the lack of adequate shaded areas and overcrowding may trigger poor welfare outcomes (i.e., pacing, and prolonged standing). However, this was a simple behavioral and preliminary study; further studies should therefore be conducted, including measurement of physical and physiological parameters, in order to confirm our results.

## Figures and Tables

**Table 1:** Ethogram used for analyzing the behavior of dromedary camels kept with and without shade at the dromedary camel market in Doha, Qatar. Behavior was analyzed using an ad libitum sampling method. Variables were measured as duration (D) or frequency (F).

Category	Behavior	Measurement Method	Definitions
Mutually exclusive (Locomotory behavior)	Walking	D	The camel does more than 2 complete steps.
	Standing	D	The camel stands on all four feet.
	Recumbency	D	The camel sits in sternal or lateral recumbency.
Non-mutually exclusive	Feeding	D	The camel takes food into its mouth (hay or concentrate), chews and swallows it.
	Rumination	D	A bolus goes back into its mouth and the camel chews it.
	Drinking	D	The camel drinks from a drinking point.
	Positive interaction	F	The camel comes into physical contact (e.g., touches, sniffs, allo-grooming) with another camel.
	Aggressive interaction	F	The camel attempts or bites/pushes or kicks another camel.
	Vocalization	F	The camel vocalizes.
	Self-grooming	D	Repeated grooming movement of mouth and incisors directed at the camel’s own body parts (e.g., scratches its head with its foot).
	Pacing in circles/on a line	D	The camel walks repeatedly from one point to another without any clear motivation.
	Bar-mouthing	F	The camel licks, bites or plays with the lips on the fence bars.
	Self-biting	D	The camel bites different parts of its own forelegs
	Head-shaking	F	The camel raises its head to the vertical (up to 90°) with a fast movement

**Table 2:** Parameters indicating the relative importance of behaviors in classifying the camel location: discriminant loadings, Wilks’ Lambda, and significance of the F test (*p*).

Behaviors	Discriminant Loadings	Wilks’ Lambda	*p*
Walking	−0.597	0.945	0.021
Recumbency	0.536	0.995	0.039
Vocalization	−0.512	0.959	0.048
Rumination	0.400	0.975	0.121
Standing	−0.235	0.991	0.359
Self-grooming	−0.167	0.995	0.515

## Data Availability

The data presented in this study are available on request from the corresponding author. The data are not publicly available due to privacy restraints, as the data set also comprises sensitive information.

## Supplementary Materials

The following are available online at https://www.mdpi.com/2076-2615/11/2/480/s1, Figure S1: Frame of a video recorded at Doha market with dromedary camels in a pen without shaded areas. The camels are resting in sternal recumbency near the fence, possibly attempting to seek passive shade; Figure S2: Lateral recumbency shown by a dromedary camel housed in a pen of 90 m2 with a shelter of 15 m2 at the camel market in Doha (Qatar); Figure S3: Frames of videos recorded at Doha market with dromedary camels in two pens: (a) a pen with a feeding point in the sun; (b) a pen with a feeding point located partly in the sun and in the shade. Red arrows indicate the feeding points; Table S1. Descriptive statistics for the behaviors noticed for each dromedary camel kept in pens with shelter (Group 1 sun and Group 1 shade) and in pens without shelter (Group 2 sun) at the camel market in Doha. SD = standard deviation; Mdn = median; IQR= interquartile range; n = number of events; s = duration of the behavior in seconds.
