# Supplementary material for: Do Camels (Camelus dromedarius) Need Shaded Areas? A Case Study of the Camel Market in Doha
Source: Animals (Basel). 2021 Feb 11;11(2):480. doi: 10.3390/ani11020480 (PMC7917598; doi:10.3390/ani11020480)
Supplement: Supplementary file 1 [file animals-11-00480-s001.pdf]

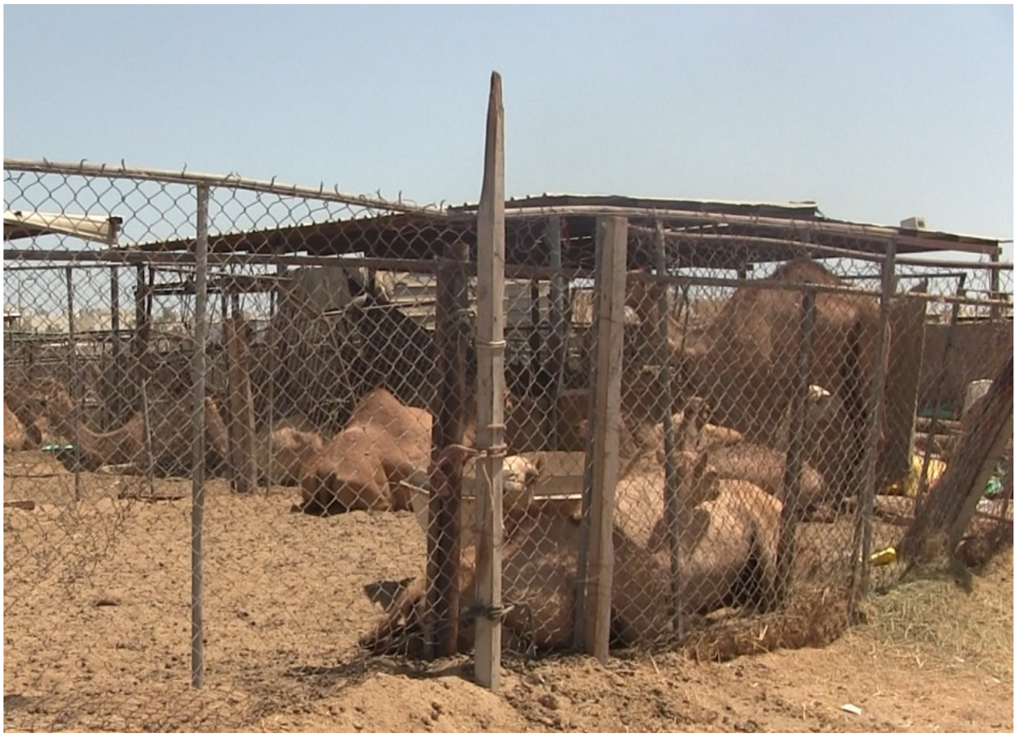

Figure S1. Frame of a video recorded at Doha market with dromedary camels in a pen without shaded areas. The camels are resting in sternal recumbency near the fence, possibly attempting to seek passive shade.

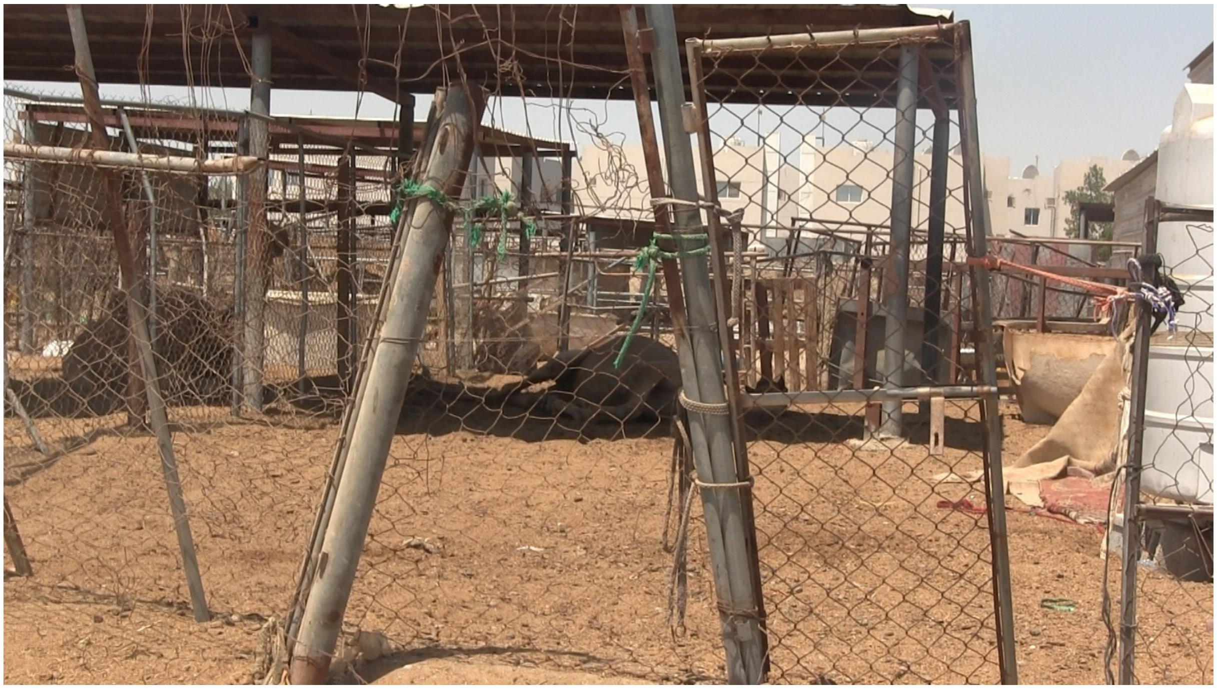

Figure S2. Lateral recumbency shown by a dromedary camel housed in a pen of 90 m^2 with a shelter of 15 m^2 at the camel market in Doha (Qatar).

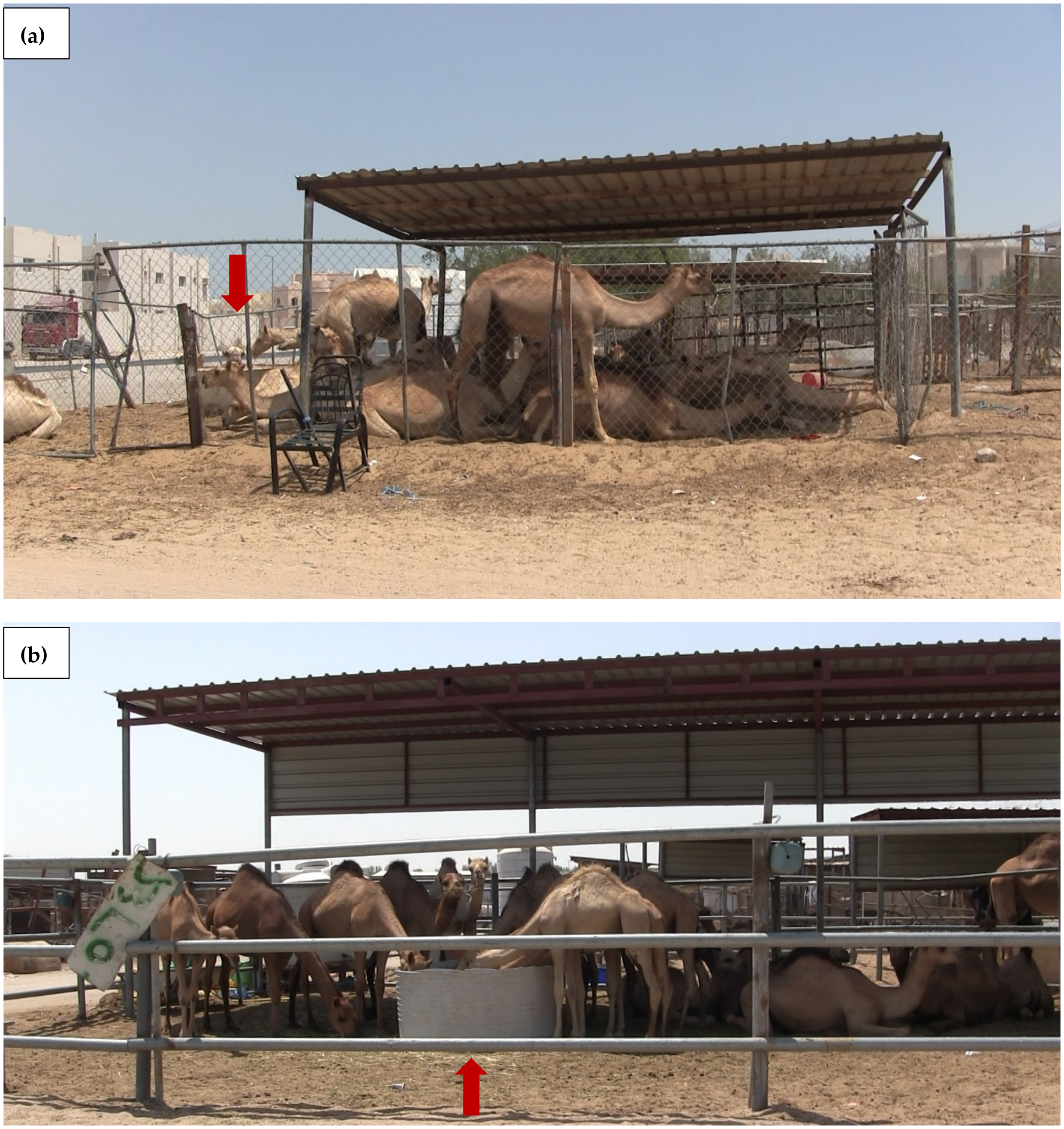

Figure S3. Frames of videos recorded at Doha market with dromedary camels in two pens: (**a**) a pen with a feeding point in the sun; (**b**) a pen with a feeding point located partly in the sun and in the shade. Red arrows indicate the feeding points.

Table S1. Descriptive statistics for the behaviors noticed for each dromedary camel kept in pens with shelter (Group 1 sun and Group 1 shade) and in pen without shelter (Group 2 sun) at the camel market in Doha. S.D. = standard deviation; Mdn = median; IQR = interquartile range; n = number of events; s = duration of the behavior in seconds.

Behaviors	Group 1 Sun		Group 1 Shade		Group 2 Sun	
	Mean±S.D.	Mdn (IQR)	Mean±S.D.	Mdn (IQR)	Mean±S.D.	Mdn (IQR)
Walking (s)	3.2 ± 6.3	0.0 (0.0–4.0)	0.6 ± 2.0	0.0 (0.0–0.0)	2.9 ± 6.3	0.0 (0.0–1.6)
Standing (s)	17.8 ± 23.1	0.0 (0.0–37.0)	15.1 ± 21.9	0.0 (0.0–27.3)	22.1 ± 25.7	10.6 (0.0–43.9)
Recumbency(s)	31.2 ± 27.9.	40.0 (0.0–60.0)	43.1 ± 23.2	60.0 (26.8–60.0)	39.8 ± 27.8	55.7 (11.7–60.0)
Feeding (s)	4.0 ± 13.7	0.0 (0.0–0.0)	3.5 ± 9.8	0.0 (0.0–0.0)	1.3 ± 3.7	0.0 (0.0–0.0)
Rumination (s)	5.9 ± 10.8	0.0 (0.0–6.1)	10.7 ± 13.9	5.3 (0.0–17.1)	6.8 ± 9.6	0.0 (0.0–14.8)
Positive interaction (n)	0.0 ± 0.1	0.0 (0.0–0.0)	0.1 ± 0.2	0.0 (0.0–0.0)	0.1 ± 0.2	0.0 (0.0–0.0)
Aggressive interaction (n)	0.0 ± 0.1	0.0 (0.0–0.0)	0.0 ± 0.0	0.0 (0.0–0.0)	0.0 ± 0.0	0.0 (0.0–0.0)
Vocalization (n)	0.1 ± 0.3	0.0 (0.0–0.0)	0.1 ± 1.1	0.0 (0.0–0.0)	0.0 ± 0.0	0.0 (0.0–0.0)
Self-grooming (s)	0.9 ± 3.7	0.0 (0.0–0.0)	0.4 ± 1.6	0.0 (0.0–0.0)	0.0 ± 0.0	0.0 (0.0–0.0)
Pacing in circles/on a line (s)	0.5 ± 2.0	0.0 (0.0–0.0)	0.5 ± 3.8	0.0 (0.0–0.0)	0.0 ± 0.0	0.0 (0.0–0.0)
